# Screening the Personal Need for the Structure and solving word problems with fractions

**DOI:** 10.1186/s40064-016-2285-3

**Published:** 2016-05-17

**Authors:** Valeria Svecova, Gabriela Pavlovicova

**Affiliations:** Constantine the Philosopher University in Nitra, Tr. A. Hlinku 1, 949 01 Nitra, Slovakia

**Keywords:** Personal Need for Structure, Fractions, Word problems

## Abstract

This paper presents the results of a pilot study of the impact of Personal Need for Structure of the selected mathematical competence. We were interested whether there is a relationship between cognitive-personal variable (Personal Need for Structure) and the mathematical knowledge about fractions of freshmen. We realized the experiment with 113 students of the Constantine the Philosopher University in Nitra, Slovakia. We examined statistically significant dependencies between the cognitive-personality variable of the Personal Need for Structure and its subfactors F1 (the wish of the structure) and F2 (the reaction to the lack of the structure) and the success rate of solving tasks and word problems with fractions. We used statistical Cochrane Q test to detect dependencies between factors of the Personal Need for Structure scale and the Mathematical knowledge. We proved that the success rate of solving word problems with fractions is inversely proportional to the need for the structure. This means that the higher the overall score on the Personal Need for Structure scale and its subfactors is, the lower is the success rate of solving the word problems.

## Background

A theoretical construct of the Personal Need for Structure (PNS) is based on the assumption of a certain individual’s ability to reduce the uncertainty of the situation (of any situation), which is associated with a greater ability to meet the new situation and cope with stressful situations. The cognitive-individual variable PNS is characterised by the representation of simplified information; by generalisation of previous experiences (Markus and Zajonc 1985; Allport 1954; Abelson 1981; Bruner 1957; Fiske and Taylor 1991) and by organising information into less complex categories which an individual uses in new and ambiguous situations to preserve or keep his/her certainty (Neuberg and Newsom 1993).

The research on PNS is mainly associated with variables like stereotypes and bias (Neuberg and Newsom 1993; Sarmány-Schuller 1999; Stangor 2000). A high need for structure is related to the need for rapid, simple and exact responses and for diverting away from uncertain or ambiguous information (Kruglanski and Webster 1996; Kruglanski et al. 2000; Webster and Kruglanski 1994). The individual feels uncomfortable and uncertain in uncertain situations (Thompson et al. 2001; Neuberg et al. 1997; Neuberg and Newsom 1993).

Neuberg and Newsom (1993) identified two conceptual different factors of the need for structure—desire for structure (F1—to have a structured environment) and response to the lack of structure (F2—an individual’s response to the lack of structure in a specific situation).

The F1 factor—*desire for the structure* is referred to as the extent to which the individuals want to establish a structure in their daily lives. People with a high desire for structure prefer the clear and structured way of life and a certain place for everything. The F2 factor—*response to the lack of structure* is referred to as the extent to which the individuals respond to unstructured, unpredictable situations. People who expressively dislike uncertain situations or changes in their plans at the last moment achieve a high score in the response to the lack of structure. The results of research studies conducted by Neuberg and Newsom (1993) indicate that both factors of the PNS scale are in different relationships to various individual features: response to lack of structure correlates with neuroticism and introversion, while desire for structure does not.

Steinmetz et al. (2011) proved that there is a stronger relationship between a rigidity scale and the F1 factor than the rigidity scale and the F2 factor. The F1 factor is mainly focused on “the need”, or personal desire for a structured and known environment (situations) while the F2 factor is focused on “the response”, or response to an unstructured and uncertain environment (situations). He also proved that the F2 factor correlates with adaptability. He reasoned this by the fact this reply is closer to the way how people “think” or how they negotiate with an unstable environment (situations). A higher personal need for structure may represent lower adaptability and flexibility. Stranovská et al. (2013) proved a negative relationship between need for structure and verbal intelligence.

Several studies have indicated that math anxiety interferes with cognitive processing via the reduction of working memory capacity (Meece et al. 1990; Ashcraft and Kirk 2001; Ashcraft 2002). Burns and Isbell (2007) proved that mathematical intelligence is dependent on a simplified categorical thinking and the proved that long thinking can stimulate the fear and increase math anxiety. This study was the impulse to analyse the extent to which individual differences in need for simple design and structure affect the success or possible failure of the processing of mathematical information.

It is important to mention that equal application of a simplified, distinctive cognitive structure to mathematical concepts and procedures may explain why some people face greater challenges than others when confronted with situations requiring effective integration and application of mathematical concept (definitions and methods).

Therefore, we also draw our attention to the impact of personal structure on the selected mathematical competence. Since this is a pilot research, we focused on “fractions”. We concentrated on the use of different algorithms and their application in solving word problems.

The rest of the paper is structured as follows. In “Theoretical framework” section we characterize cognitive-individual variables *Personal Need for Structure* and *Mathematical knowledge. In this section*, *we also* refer to similar studies about these variables. In section three we describe methods, we used within the research, and we outline the aims of the study. A summary of the research outcomes and the analysis of the variables influence on syntactic abilities can be found in section four. Discussion and conclusion are presented in the last part.

## Theoretical framework

Based on Sternberg (2002), cognitive psychologists are examining the biological basis of:

- cognitive processes,
- attention,
- consciousness,
- perception,
- memory,
- mental representation,
- language,
- problem solving,
- creativity,
- decision making,
- cognitive developmental changes throughout life,
- intelligence, artificial intelligence and many other aspects of thinking.

The ability to develop cognitive structure can be presented as the measure how an individual is able to avoid information, which does not conform the structure of knowledge he already has.

The ability to develop cognitive structure refers to the way individuals (and the group) perceive and process information about uncertain incentives and uncertain situations or situations that are considered as uncertain by specific people. As reported by Bar-Tal and Spitzer (1999) high capability of creating cognitive structures is related to the ability to solve new ambiguous situations, the willingness to experiment with the unknown, the desire to change established ways of problem solving, to act in social situations using heuristic methods effortlessly.

### Personal Need for Structure

Several psychologists (Abelson 1981; Markus and Zajonc 1985; Fiske and Taylor 1991; Neuberg and Newsom 1993) dealt with cognitive-personal variable PNS. They found that this variable is characterized by a representation of simplified information, generalization of previous experience, an organization of information into fewer complex categories that the individual uses in new and ambiguous situations in order to maintain security.

Research results of Sarmány-Schuller (1999) pointed out that the need for the structure has significant positive correlation with the preference for abstract conceptualization. This means that people with high need for the structure have problems with “active experimentation” (willingness to cross their borders, willingness to change their established ways of behaviour, thinking, attitudes, simple structure). Research of PNS is associated with variables such as stereotypes and prejudices. The high personal need for structure is characterized by the need for fast, easy and accurate answers, a step away from uncertain or ambiguous information. In uncertain situations, the individual feels uncomfortable and insecure.

The primary purpose of our research was to bridge previous research findings on social stereotyping with further exploration into math anxious students’ maladaptive modes of integrating information.

The aim of the research is to determine the relationship between the F1, F2 factors of PNS and the process of solving fraction tasks. We used existing certified methods of measuring the need for a structure in the field of psychology of personality. Current international studies suggest the need to apply these methods when exploring the development of mathematical powers. This seems to be quite a weak section on the scale used in Slovakia.

### Personal Need for Structure and mathematics

In the year 2013 in England, research focused on the impact of mathematics anxiety and personal structure of mathematical thinking was made. There were 99 participants, which consist of 53 students of mathematics and 46 students of psychology. The primary hypothesis driving this experiment was that when respondents were confronted with mathematical content, math anxious respondents would demonstrate the need to impose inappropriate and maladaptive broad categorical thinking. This hypothesis was stated based on the review of social cognition literature that focuses on the cognitive processes that underlie stereotyping in social realms. Furthermore, for those who adopted an entity theory, it is predicted the have high math anxiety and the need for simple structure, respectively. When we take together the literature on anxiety, social stereotyping and implicit theories of intelligence, we can better understand the specific cognitive responses to the complexity that may underlie barriers to effective mathematical processing and computation. This research also tried to explain the impact of presenting complex mathematical concepts through a series of incremental steps designed to address the cognitive and emotional needs of math anxious students, while at the same time promoting the development of valid mathematical reasoning abilities (Sarnataro-Smart 2013).

### Mathematical knowledge

The current Slovak curriculum puts into the center of mathematical education at the primary level of primary school acquaintance with natural numbers and with four basic mathematical operations. Later, the field of natural numbers expands in two directions: *fractions and decimal numbers* and *negative numbers*.

Natural numbers, which represents an amount, are relatively easy to understand and to imagine. We can count and say, e.g. how many apples we have in the bowl. However, fractions are a problem for many people as they concern the relations between the quantities. What is a half? One-half of what? If Alice and Bob spent their pocket money on food, it does not mean that they spent the same amount of money.

According to Hejný (2004), problems of students with fractions leads mathematics teachers to divide mathematics education into several areas, based on answers to following questions:

- How to open the world of fractions for students who want to participate?
- What are the causes that students do not understand fractions?
- How can the situation be improved?

Mathematics teachers firstly tried to find answers to these questions in theories of the separate and generic model. Later, they used other theories, especially the theory of reification from Sfard [as cited in Hejný (2004)]. The skeleton of this theory is a sequence of five stages:

Processes of objects → Interiorizing → Condensation → Reification → New object

At the beginning of this theory, the activities of younger students are mostly manipulative. Records of them are stored in the memory of the student as experiences, which are interiorized in a meaning of Piaget’s theory (the pupil interiorizes not only activities but also their products, and he can equip them again in his imagination). Achieved experiences are often various; they are connected to each other, and they are condensing into one organic whole, which changes into preterm and term. This last step describes Sfard as condensed experience.

Between the theory of reification and the model theory are many intersections, e.g. Sfard process of the objects corresponds to the phase of separate models and condensation, to the reification we can often assign a phase of separated models and condensation, and to the reification we can assign a phase of generic models. The theory of reification emphasizes dynamic phenomena and theory models emphasize static phenomena. These two approaches fill up each other.

## Research

According to Grebenev et al. (2014), physical and mathematical education traditionally raises specific and strict demands for the cognitive sphere of students. These requirements are determined by a combination of inductive and deductive stages of the educational process, a study of logically complete copies of the physical theories, and a large share of independent practical exercises.

Psychodidactical aspects present the teacher as mediator of student’s knowledge. Therefore, it is not just a process to give the topic from the teacher to the pupil, but the emphasis on the pupil’s personality also increases, as well as the focus on his cognitive development and learning and personal characteristics. According to Maj (2008), it is necessary that the competency of the teacher go beyond the union and didactic knowledge, as well as beyond pedagogical skills. These competencies should go psychological direction too, both regarding pupils’ knowledge and their way of thinking, as well as regarding their personal development.

The research of mathematical anxiety of secondary school students and older led to the experiences and memories of teaching elementary mathematics at younger school age. Therefore, we chose primary school teachers in service as participants in our research. The selection of education methods and teaching forms in the academic praxis is influenced by teachers’ teaching competencies and professional competence as well. It also influences his/her internal attitude for an individual subject, which he/her consciously or unconsciously transfers to the students. So when the teacher is not able to apply mathematical knowledge and skills on an informal level (to solve open or application tasks) he/her tends to avoid such kind of the tasks in the teaching process. That means, if the mentioned tasks occur in the worksheets, the teacher tends to omit them (if the national program of education permit it).

The results of previous research, as we describe in more detail in Švecová (2015), showed that the need for structure (sub-factor F1—desire for structure) is related to knowledge preference for the multiplication operation and to algorithm converting the mixed number to a fraction, namely a statistically significant negative correlation between desire for structure and knowledge preference for the multiplication operation (r = −2.2) and statistically significant negative correlation between *desire for structure* and *converting the mixed number to a fraction* (*r* = −1.6).

We chose the theme of fractions because we assumed that the individual algorithms for operations with fractions provides a suitable environment, which can be seen as a structure. The knowledge of the various mathematical algorithms may evoke in students certain confidence when they work with fractions. Our assumptions have been verified by experience from the real life. Students can quite quickly learn algorithms for summation, subtraction, multiplying and division. Thus, they are capable of performing arithmetic operations with fractions. According to Tichá and Macháčková (2006), the older a student is, the more he prefers templates. The students tend to leave the visual representation, solving by deduction or using trial-and-error method, and they shift towards the symbolic representations. They use mainly equations.

### Methods

We were interested whether there is a relationship between the personal structure and the mathematical knowledge about fractions of freshmen. To determine the appropriate structure, we used the PNS, and to detect dependencies between factors of the PNS and Mathematical knowledge was used statistical Cochrane Q test.

#### The PNS scale

The PNS scale is based on the assumption that the ability to reduce the uncertainty of the situation is bound to the capacity to deal with the new situation. The scale determines the tolerance of students for the uncertainty of situations in mathematics.

The PNS construct is based on a two-factor concept of personal need for structure (Sarmány-Schuller 1999):

1. The desire for structure (sub-factor F1).
2. The response to the lack of structure (sub-factor F2).

The total score of the personal need for structure PNS is obtained as a sum of scores for both sub-factors. The reliability analysis conducted on the PNS scale yielded a Cronbach’s alpha of 0.88, with the item- total correlation between 0.58 and 0.60 (Moskowitz 2011). Table 1 contains factor loadings, reliability analyses of the PNS scale and items of PNS scale.

**Table 1:** Factor loadings and reliability analyses of the PNS scale (Moskowitz 2011, p. 26)

Item	Factor loading	Item-total correlation
1. It upsets me to go into a situation without knowing what I can expect from it	0.56	0.45
2. I am not bothered by things that upset my daily routine	0.59	0.48
3. I enjoy having a clear and structured mode of life	0.66	0.57
4. I like a place for everything and everything in its place	0.61	0.51
5. I like being spontaneous	0.54	0.44
6. I find that a well-ordered life with regular hours makes my life tedious	0.61	0.50
7. I do not like situations that are uncertain	0.58	0.48
8. I hate to change my plans at the last minute	0.68	0.58
9. I hate to be with people that are unpredictable	0.57	0.47
10. I find that a consistent routine enables me to enjoy life more	0.69	0.60
11. I enjoy the exhilaration of being put in unpredictable situations	0.64	0.53
12. I become uncomfortable when the rules in a situation are not clear	0.55	0.43
Eigenvalue		4.45
Total% variance		37.8
Cronbach’s alpha		0.84

#### The mathematical test

The mathematical test was designed according to ISCED 2 for external testing of pupils in ninth grade (15 years) of primary school. This mathematical test is in Slovakia carried annually in order to detect the individual level of pupils’ knowledge of Mathematics. We selected word problems with fractions from these testing. The test included five word problems. The example of one of these word problems is as follows:

In a company of 1050 employees are 2/3 women. 4/5 women have a professional qualification. Calculate: (1) How many women work in the company? (2) How many women do not have a professional qualification? (3) How many men work in the company?

#### Cochran’s Q test

We assume a negative correlation between the personal need for structure and solving word problems with fractions. Cochran’s Q test verified this assumption. Cochran’s Q test is an extension to the McNemar test for related samples that provides a method for testing for differences between three or more matched sets of frequencies or proportions. This is an alternative to the one-way analysis of variance for repeated measures (Munk 2011).

#### Participant

The participants in our research were 113 university students in Preschool and Elementary Education at the Constantine the Philosopher University in Nitra. After passing the Bachelor’s degree, most of the students in Preschool and Elementary Education continue to the Master’s degree in the study program The Teacher Training for Primary Education. Later, these students will be teachers for primary level of elementary education, where mathematics plays an important role. The average age of the students was 20.5 years. The summary of their graduated secondary schools is in Table 2.

**Table 2:** Distribution of students by type of high school they graduated from

Type of school	Number of students
High school	28
Pedagogical and social academy	36
Business academy	20
Another grammar school	29

As we can see in Table 2, most of the students have undergone grammar school with pedagogical direction, what is related to the chosen study program of Preschool and Elementary Education.

In the first phase of the research, the participants completed the PNS scale and also mathematic test focused on the application of algorithms when computing the fractions. In the second phase, participants solved the word problems with fractions. The duration of the mathematic test was 30 min. We examined the correlation between the personal need for structure and resolve the word problems with fractions in this paper.

## Evaluation and discussion

We assume that there is a negative correlation between the *personal need for structure* and *solving word problems with fractions.* We stated following hypothesis verified by using non-parametric tests of the mean value.

### **H0**

The values of variables in a vector of variables F1 and F2 do not depend on solving word problems with fractions.

Students solved five word problems with fractions. An example of one of these word problems was in the previous section. The solution of problems consisted of three steps.

- The first step (Step 1) was to determine (calculate) a part of the whole. Fifty-two students solved this part of the task correctly; that represents approximately 46 %.
- It was necessary to use two arithmetic operations to solve the Step 2 and there were two possible solution strategies. The first solution strategy (Step 2a) was to determine the part of the whole and then subtract the result from the whole. Twenty-five students correctly used this strategy; it is approximately 22 %. The most often mistake was that students calculated only part of the whole and they considered it as the correct result. The second strategy was to determine the remaining part of the whole (Step 2b). We consider this second strategy as more demanding as it requires a different view on the problem. Eleven students solved the task by using this strategy, which represents almost 10 %. This step caused the biggest problem for the students.
- The third step (Step 3) is based on Step 1, and just the subtraction was needed. Those students, who correctly made Step 1, made Step 3 correctly too.

The given word problem was correctly solved by 34 students, which is 30 %. This low success rate is probably related with the major impediment, which Hejný (2003) refers to the inability of students to understand the word problem, to understand the situation described by the word problem and/or to understand the challenge of the word problem.

Scheffe test for multiple comparisons confirms the various difficulty of each step (Table 3). The individual steps of the word problem created two homogeneous groups. The first homogeneous group consists of Step 2a and Step 2b. The second homogeneous group consisted of Step 1 and Step 3. Test for multiple comparisons confirmed that there is a statistically significant difference between these two groups. This is also evident from the fact that student who correctly solved the problem using Step 1 and Step 3 may be wrong in Step 2. When students wrongly designed Step 1, they were also wrong in Step 2.

**Table 3:** Scheffe test for multiple comparisons confirms the various difficulty of each step

Step	1	2
(2b)	****	
(2a)	****	
3		****
1		****

We were interested, if and how the individual steps of the solution of given word problem are related to the personal needs of the structure and its subfactors F1 and F2. The results of non-parametric correlation between different variables are presented in Table 4.

**Table 4:** Dependence between variables PNS, F1, F2 and the steps of the word problem

	γ	Z	p value
Step 1 and PNS	−0.202096	−2.20315	0.027584*
Step 1 and F1	−0.162838	−1.75124	0.079905
Step 1 and F2	−0.218315	−2.36709	0.017928*
Step (2a) and PNS	−0.320169	−2.91986	0.003502*
Step (2a) and F1	−0.235694	−2.12026	0.033984*
Step (2a) and F2	−0.327924	−2.96757	0.003002*
Step (2b) and PNS	−0.088889	−0.57637	0.564366
Step (2b) and F1	−0.156993	−1.00798	0.313464
Step (2b) and F2	−0.092764	−0.60487	0.545268
Step 3 and PNS	−0.214941	−2.34241	0.019160*
Step 3 and F1	−0.168076	−1.80574	0.070959
Step 3 and F2	−0.235255	−2.55416	0.010645*

* p value < 0.05

Table 4 illustrates the significant differences at 0.05 level of significance between the variable PNS, subfactor F2 and the Step 1 (number of women). Furthermore, we proved statistically significant differences between the Step 2a and PNS variable (significant level 0.01), as well as between Step 2a and both factors F1, F2 (significant level 0.01). There is also statistically significant difference between variables PNS, F2 and the Step 3 (number of men). That means that we reject the null hypothesis H0, which claims that there is no statistically significant difference between variables PNS, F1 and F2 and the solution of the word problem with fractions with 95 % reliability. So we can conclude that there is an inversely significant dependence between the variable F1 and solving word problems with fractions. It can be concluded that the higher is the total score of the PNS scale and its factors F1 and F2; the lower is the percentage of solving word problems. We assume that this situation can be related to the fact, that solving the word problems is not a sufficiently structured environment for students with a high score in PNS.

**Fig. 1:**
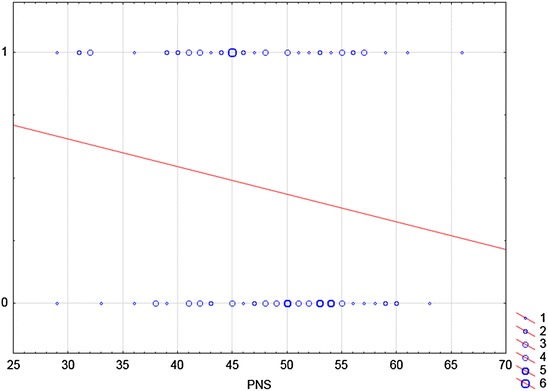
The relationship between variable PNS and Step 1 of word problem. This figure is a graph of logistic regression. Logistic regression is this a mathematical modelling approach in which the best-fitting, yet least-restrictive model is desired to describe the relationship between several independent explanatory variables and a dependent dichotomous response variable. In our case, a *straight line* reflects the probability that respondent resolved the Step 1 of a problem if the PNS scale reached a certain score. In Step 1 respondents determined (calculated) part of the whole. *X-axis* displays the independent variable. It is a score achieved by individual respondents in a PNS scale. The *y-axis* shows the dependent variable of interest. One stands for the correct solution, zero for the incorrect solution. The *blue circles* represent the number of respondents who reached a certain score on the PNS scale and who were successful/unsuccessful in Step 1 of the problem. For example, six students who reached 45 points on the PNS scale, correctly solved the Step 1. On the contrary, five of the respondents who were unsuccessful in Step 1, had 50 points on the PNS scale

**Fig. 2:**
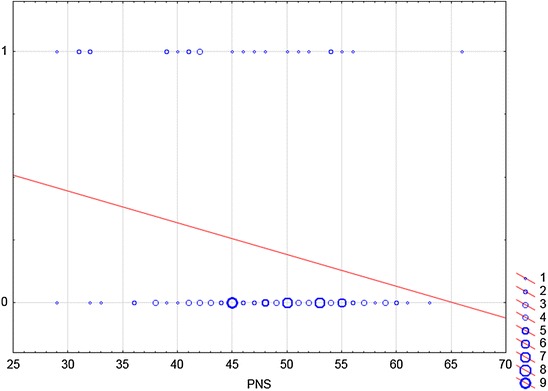
The relationship between variable PNS and Step (2a) of word problem. This figure is a graph of logistic regression. Logistic regression is this a mathematical modelling approach in which the best-fitting, yet least-restrictive model is desired to describe the relationship between several independent explanatory variables and a dependent dichotomous response variable. In our case, a *straight line* reflects the probability that respondent resolved the Step 2 of the task if the PNS scale reached a certain score. During the solving process in Step 2, it was necessary to use two arithmetic operations. The first solution strategy was to determine part of the whole and subtract the result from the whole. *X-axis* displays the independent variable. It is a score achieved by individual respondents in a PNS scale. The *y-axis* shows the dependent variable of interest. One stands for the correct solution, zero for the incorrect solution. The *blue circles* represent the number of respondents who reached a certain score on the PNS scale and who were successful/unsuccessful in Step 2 of the problem solving. For example, four students who reached 42 points on the PNS scale, correctly solved the Step 2. On the contrary, nine of the respondents who were unsuccessful in Step 2, had 50 points on the scale PNS

**Fig. 3:**
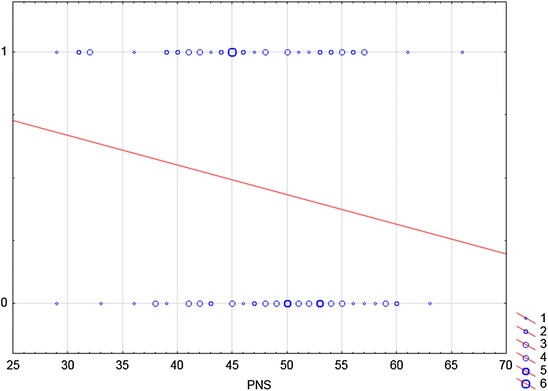
The relationship between variable PNS and Step 3 of word problem. This figure is a graph of logistic regression. Logistic regression is this a mathematical modelling approach in which the best-fitting, yet least-restrictive model is desired to describe the relationship between several independent explanatory variables and a dependent dichotomous response variable. In our case, a straight line reflects the probability that respondent resolved the Step 3 of a problem if the PNS scale reached a certain score. In Step 3 which is based on Step 1, just the subtraction operation was needed. *X-axis* displays the independent variable. It is a score achieved by individual respondents in a PNS scale. The *y-axis* shows the dependent variable of interest. One stands for the correct solution, zero for the incorrect solution. The *blue circles* represent the number of respondents who reached a certain score on the PNS scale and who were successful/unsuccessful in Step 3 of the problem. For example, six students who reached 45 points on the PNS scale, correctly solved the Step 3. On the contrary, four of the respondents who were unsuccessful in Step 3, had 55 points on the scale PNS

Figures 1, 2 and 3 represent graphs of logistic regression. Logistic regression is this a mathematical modelling approach in which the best-fitting, yet least-restrictive model is desired to describe the relationship between several independent explanatory variables and a dependent dichotomous response variable. In our case, a straight line reflects the probability that respondent resolved the step of the task if the scale PNS reached a certain score.

We accept the argument that success in solving the word problem has an inverse connection with the needs of the structure. The word problems could cause a difficult situation, stress, and uncertainty for students with a high personal need for structure. As reported by John et al. (2000) uncertainty cannot be omitted from the learning process, but its impact can be examined and to what measure can be this impact minimalized. This fact will be in the focus of our next interest. Brown (2000) recommends to teach individuals toward tolerance for ambiguity (intuitive behaviour, trial-and-error, to not search for rules and algorithm for each time) and to accept it as a part of learning.

## Conclusion

In conclusion, we can say that the word problems and their solution are a significant variable in our research that influences needs of the structure and success in mathematics. The impact of personal needs of the structure on the mathematic competence is an area of the research that is not very explored yet, but it may affect achievements of the student in mathematics. Similar studies in the field of psycholinguistics were taken by Munková et al. (2014).

We prove that the success of solutions to word problems with fractions depends inversely on the need for structure, meaning that the higher the total score of the PNS and its subfactors F1 and F2, the lower the success rate in solving word problems. Based on the results of statistical processing of the claim we note that the percentage solutions to word problems with fractions depend inversely on the need for structure. This fact corresponds with the claim that word problems represent for students with high scores PNS inadequately structured environment. Thus, word problems as such are for students with high needs for structure stressful and raise uncertainty in some situations.

We agree with the research results of Fujimoto et al. (2005), Bouckenooghe et al. (2007) which showed that the need for a structure is connected with the ability to solve new ambiguous situations that mean to change the usual ways of behaviour, thinking, attitudes, and simple structures.

We can conclude that word tasks and their solutions appear in our research as an important variable which influences the need for structure and success rate in mathematics. The effect of the personal need of a structure on mathematical powers is a part of the research that has not been explored very much so far but which can significantly affect results the students achieve in mathematics.

We see the future direction of research in the analysis of the need for structure in other areas of mathematics, geometry including. As already mentioned repeatedly percentage of pupils/students in solving problems may be related to the mathematical anxiety. Therefore, we want to focus attention on the analysis of the impact of the necessary structures for math anxiety. This also includes the creation of an intervention or program in mathematics education.

## Abbreviations

PNS: Personal Need for Structure
F1: PNS factor desire for the structure
F2: PNS factor response to the lack of structure
